# Do Mismatches between Pre- and Post-Natal Environments Influence Adult Physiological Functioning?

**DOI:** 10.1371/journal.pone.0086953

**Published:** 2014-01-31

**Authors:** Tony Robertson, Michaela Benzeval

**Affiliations:** 1 Scottish Collaboration for Public Health Research & Policy, University of Edinburgh, Edinburgh, United Kingdom; 2 Institute for Social & Economic Research, University of Essex, Colchester, Essex, United Kingdom; 3 Medical Research Council/Chief Scientist Office Social and Public Health Sciences Unit, University of Glasgow (Visiting Professor), Glasgow, United Kingdom; CUNY, United States of America

## Abstract

**Purpose:**

Mismatches between pre- and post-natal environments have implications for disease in adulthood. However, less is known about how this mismatch can affect physiological systems more generally, especially at younger ages. We hypothesised that mismatches between pre- and post-natal environments, as measured by the measures of birthweight and adult leg length, would be associated with poorer biomarker levels across five key physiological systems in young adults.

**Methods:**

Data were collected from 923, 36 year-old respondents from the West of Scotland Twenty-07 Study. The biomarkers were: systolic blood pressure (sBP); forced expiratory volume (FEV_1_); glycated haemoglobin (HbA_1c_); glomerular filtration rate (eGFR); and gamma-glutamyltransferase (GGT). These biomarkers were regressed against pre-natal conditions (birthweight), post-natal conditions (leg length) and the interaction between pre- and post-natal measures. Sex, childhood socioeconomic position and adult lifestyle characteristics were adjusted for as potential effect modifiers and confounders, respectively.

**Results:**

There were no associations between birthweight and leg length and sBP, FEV_1_, HbA_1c_, or GGT. Higher birthweight and longer leg length were associated with better kidney function (eGFR). However, there was no evidence for mismatches between birthweight and leg length to be associated with worse sBP, FEV_1_, HbA_1c_, eGFR or GGT levels (*P*>0.05).

**Conclusions:**

Our hypothesis that early signs of physiological damage would be present in young adults given mismatches in childhood environments, as measured by growth markers, was not proven. This lack of association could be because age 36 is too young to identify significant trends for future health, or the associations simply not being present.

## Introduction

The foetal origins of disease have been widely studied, with pre-natal growth (often measured through birthweight and used as a marker for the uterine developmental environment) consistently linked with the later development of adult disease [1]. However, there is mounting evidence that the association between pre-natal development and later health is strengthened when post-natal conditions are considered. Typically, poorer growth *in utero* (as a result of, for example, poor nutrition, maternal smoking/alcohol consumption or maternal stress) [2] followed by rapid growth in childhood (as a result of, for example, increased nutrition, reduced stress or decreased infections) [3] appears to have the greatest detrimental effect on health [4]–[7]. Although there is increasing evidence of the implications for disease in adulthood following such plasticity in growth brought about by mismatches in environmental circumstances, less is known about how mismatches could affect the physiological systems in the body more generally, especially at younger ages. This relationship is important as it could improve not only our understanding of the early-life influences on disease process, but also enable earlier intervention prior to the onset of disease.

The aim of this study was to examine the influence of mismatches between pre- and post-natal conditions across five key physiological systems important to health and functioning. The biomarkers selected were: systolic blood pressure (sBP; circulatory system); forced expiratory volume in one second (FEV_1_; respiratory system/lung function); glycated haemoglobin (HbA_1c_; metabolic system/diabetes marker); estimated glomerular filtration rate (eGFR; excretory system/kidney function); and gamma-glutamyltransferase (GGT; hepatic system/liver function). We chose to focus on young adults to identify effects prior to the onset of disease.

Our hypothesis was that mismatches between birthweight and leg length (marker of post-natal childhood environment and growth [8]–[10]) would be associated with more negative biomarker outcomes, with the association strongest with GGT given that the liver is one of the most plastic organs after birth [5]. There is evidence that post-natal conditions continue to influence sBP [11], [12] and HbA_1c_ [11], [13]. We predicted that there would be an association between those respondents showing mismatches between pre- and post-natal conditions and these two biomarkers, although weaker than that seen with GGT (due to their reduced plasticity in childhood). The evidence for an association between mismatches between pre- and post-natal conditions and FEV_1_ is weaker than for BP and HbA_1c_ [14], [15]. Therefore it was predicted that mismatches between pre- and post-natal conditions would be only weakly associated with FEV_1_. It was predicted that eGFR would not be associated with mismatches between pre- and post-natal conditions given that kidney development is complete prior to birth [16], [17].

## Methods

### Study Sample

The West of Scotland Twenty-07 Study is a community-based, prospective cohort study started in 1987 and completed in 2007/8. Individuals were recruited using a clustered randomised sample design in the greater Glasgow area in the West of Scotland, UK. Respondents were aged 36 years at blood sampling (wave 5 of data collection). The baseline sample consisted of 1515 respondents (777 women). Due to attrition, the sample size by wave 5 was 923 (505 women). Data were collected by trained nurses in participants’ homes. More detail about the study is available elsewhere [18].

### Main Outcome Measures

Systolic BP (mmHg) was measured using a sphygmomanometer (Omron Healthcare UK Ltd., West Sussex, UK). The mean values of two readings from the same arm were used.

FEV_1_ (litres/second) was measured using a vitalograph (Micro Plus Spirometer MS03; Micro Medical, Rochester, UK). The highest value of three readings was used, standardised by dividing by height squared [19]. Serum creatinine (µmol/l) was measured using the Kinetic Jaffe method (list number 7D64, Abbott Diagnostics, Abbott, Illinois, USA). Creatinine was standardised to produce eGFR (ml/min) using the Cockcroft-Gault method [20]. Serum GGT (units/litre) was measured using the carboxyl nitroanilide (IFCC) method (list number 7D65, Abbott Diagnostics). Whole blood HbA_1c_ (%) was measured using an HPLC auto-analyser method (HA-8160; Menarini, Rungis, France). In analyses using BP and HbA1c, we identified the need to employ adjustments to the statistical models to address potential modifying effects of medications on biomarkers. For those respondents using anti-hypertensive (n = 24) or anti-diabetes (n = 7) medications we included dummy variables for medication use in the statistical models [21] [22]. As a sensitivity test, we also excluded these individuals from the analysis. The results were unchanged.

### Growth Measures

The parents of respondents were asked at baseline to recall the birthweight (kg) of the respondent. Leg length was derived from subtracting sitting height (cm) from standing height (cm) at age 36, both measured using a Seca Leicester stadiometer (Cranlea, Birmingham, UK). Each growth marker was standardised using sex-specific z-scores prior to analysis.

### Lifestyle Factors and Socioeconomic Position

Lifestyle factors can alter the physiology and biomarker levels of people throughout their lives. Given this potential for confounding the association between early-life factors and later biomarker measures, we chose to adjust for alcohol and smoking consumption, as well as obesity and physical activity. Data on alcohol consumption (units/week), smoking status (‘current’, ‘ex’ or ‘never’ smoker), Body Mass Index (BMI) and physical activity (hours of moderate or vigorous activity/week) were collected at wave 5.

Childhood socioeconomic position (SEP) is linked with both birthweight and growth in childhood, with lower SEP children showing lower birthweight [23], but greater levels of compensatory/catch-up growth in early childhood [24]. In addition, SEP is strongly linked with disparities in adult health and physiological functioning [25]. Hence, we also investigated childhood SEP as a potential effect modifier through stratified analysis, using occupation-based social class at birth (Registrar General’s Social Class). The highest ranking occupation between the two parents was selected.

### Statistical Analysis

A total of 768 respondents had complete data for birthweight, leg length, parental occupation and the lifestyle characteristics. All 768 respondents had their blood pressure recorded, but only 749 had FEV_1_ measured. 644 respondents provided blood samples suitable for measurement of HbA_1c_, with 643 providing eGFR and GGT results. The total amount of missing data across all measures was 6% for those taking part in wave 5 and 31% when all those respondents who were in the study at baseline were considered. Multiple imputation (MI) was used to address potential biases arising from missing data values. Twenty five imputed datasets were created. Imputation and analyses were performed using the ‘ice’ and ‘mim’ packages in Stata (ver.11, Stata Corp., Texas, USA). In addition to all variables used in the analyses, auxiliary variables (those not included in the analysis, but which help predict missingness) were also included in the imputation model. These included ‘any chronic illness’ and ‘self-rated health’ at wave 5, and ‘mother’s religion’, ‘whether breastfed’, ‘any birth complications’ and ‘any limiting longstanding illness in the household’ at wave 1. Linear regression was used for the statistical analyses, with all analyses adjusted for clustered sampling at baseline and were weighted to the living baseline sample at the time of the wave 5 interviews using inverse probability weights to correct for bias due to drop out [26]. These weights were also included in the imputation model. Separate analyses were run for each biomarker. For clearer interpretation of the results, higher levels of all five biomarkers reflect poorer functioning. For FEV_1_ and eGFR, results were multiplied by −1 prior to regression analysis to reflect this. The initial (‘unadjusted’) models included: birthweight, leg length and birthweight*leg length (used to assess the mismatch between pre- and post-natal growth measures [27]), all assessed as continuous variables; sex; and birth status (singleton or twin). The ‘adjusted’ models additionally included the adult lifestyle factors. The different lifestyle confounders were tested separately, although only the fully adjusted models (all confounders included) are presented due to a lack of substantive differences compared to the single-confounder models. Sub-sample analyses, stratifying by sex and SEP, were also run to investigate possible effect modification, but again showed no substantive differences compared to the full models. All analyses were weighted to the living baseline sample at the time of the wave 5 interviews using inverse probability weights to correct for bias due to drop out [26].

### Sensitivity Analysis

Categorical versions of birthweight and leg length were also considered in order to capture more extreme cases of the mismatch between pre- and post-natal growth. Birthweight was categorised as low (<2.5 kg), average (2.5–3.9 kg) or high (>3.9 kg), while leg length was converted into sex-specific tertiles. Regression analyses using a nine-category variable were employed, with those showing low birthweight and long leg length used as the reference category.

### Ethical Approval

Ethical approval for the baseline study was granted in 1986 by the GP Sub-Committee of Greater Glasgow Health Board and the ethics sub-committee of the West of Scotland Area Medical Committees. Wave 5 was approved by the Tayside Committee on Medical Research Ethics. Informed, written consent was obtained from all respondents at each wave of the study. At wave 1 (when aged 15), written consent was obtained from parents/guardians and the respondents.

## Results

The characteristics of the respondents sampled are shown in Table 1. Birthweights were comparable to the national average in 1985 [28], as were lifestyle factors compared to national data from 2011 [29]. Biomarker values were largely below clinically-relevant cut-offs. Analysis samples were similar to the complete wave 5 sample in terms of proportions within each category and mean values (not presented). The results of the analyses are presented in Table 2, where a significant negative coefficient represents a scenario where small birthweight would have a greater effect only in those with longer leg length [27]. A significant positive interaction would suggest that increasing birthweight would have a greater effect only in those with longer leg length. However, there was no evidence that mismatches between pre- and post-natal conditions were associated with any of the five biomarkers, either before or after adjustment for adult lifestyle factors (Table 2). Complete-case analysis results did not reveal any substantive differences to those presented using MI (Table S1 in File S1). Analysis of the individual growth markers (birthweight and leg length) also failed to show any associations with sBP, FEV_1_, HbA_1c_ or GGT. However, higher birthweight was associated with better kidney function (eGFR) (β = −5.45, 95% CI: −8.53, −2.38), with this association remaining after adjustment for lifestyle confounders (β = −2.95, 95% CI: −5.04, −0.87). Greater leg length was also associated with better kidney function, but only after adjustments (β = −5.35, 95% CI: −7.89, −2.81). The results of the sex and SEP-stratified analyses were no different from the full analyses.

**Table 1:** Study characteristics based on those taking part in wave 5 (non-imputed).

	Samplesize	Mean (SD)
**Sex**	**923**	
Male	418 (45.3%)	
Female	505 (54.7%)	
*Missing*	*0 (0%)*	
**Birthweight (kg)**	**877**	3.31 (0.60)
*Missing*	*46 (5.0%)*	
**Leg length (cm)**	**906**	79.52 (6.10)
*Missing*	*17 (1.8%)*	
**sBP (mmHg)**	**917**	124.45 (15.87)
*Missing*	*6 (0.7%)*	
**FEV_1_ (litres/sec)/height^2^**	**879**	1.18 (0.19)
*Missing*	*44 (4.8%)*	
**HbA_1c_ (%)**	**757**	5.21 (0.49)
*Missing*	*166 (18.0%)*	
**eGFR (ml/minute)**	**752**	116.00 (28.82)
*Missing*	*171 (18.5%)*	
**GGT (units/litre)**	**756**	35.46 (39.76)
*Missing*	*167 (18.1%)*	
**Weekly alcohol consumption** *	**848**	
Not heavy drinker	592 (62.9%)	
Heavy drinker	256 (27.2%)	
*Missing*	*93 (9.9%)*	
**Smoking status**	**918**	
Never smoked	443 (47.1%)	
Ex-smoker	217 (23.1%)	
Current smoker	258 (27.4%)	
*Missing*	*23 (2.4%)*	
**BMI**	**896**	
Underweight (<18.50)	12 (1.3%)	
Normal range (18.50–24.99)	278 (29.5%)	
Overweight (25.00–27.49)	356 (37.8%)	
Obese (≥30.00)	250 (26.6%)	
*Missing*	*45 (4.8%)*	
**Weekly physical activity** **	**918**	
Below recommended levels*	556 (59.1%)	
Meet or surpass recommended levels*	362 (38.5%)	
*Missing*	*23 (2.4%)*	
**Social class at birth (SEP)**	**892**	
I (Professional etc. occupations)	84 (9.1%)	
II (Managerial and technical occupations)	184 (19.9%)	
III – NM (Non-manual skilled occupations)	345 (37.4%)	
III – M (Manual skilled occupations)	178 (19.3%)	
IV (Partly-skilled occupations)	92 (10.0%)	
V (Unskilled occupations)	9 (1.0%)	
*Missing*	*31 (3.4%)*	

* Heavy drinker = more than 14 (women) or 21 (men) units per week.

** Based on the UK Departments of Health recommendations of 2.5 hours of moderate or 1.25 hours of vigorous activity per week for 18–64 year olds.

**Table 2:** Regression results for the five biomarkers modelled against birthweight and leg length.

		Multiple imputation analysis (n = 923)			
		Unadjusted		Adjusted	
		β	95% CI	β	95% CI
BP	- Birth weight	−0.10	−1.12, 0.92	−0.57	−1.58, 0.42
	- Leg length	−0.35	−1.94, 1.25	0.66	−0.74, 2.07
	- Birth weight*Leg length	0.41	−0.49, 1.31	0.14	−0.66, 0.94
FEV_1_	- Birth weight	−0.02	−0.03, 0.01	−0.01	−0.03, 0.01
	- Leg length	−0.01	−0.02, 0.02	0.01	−0.01, 0.03
	- Birth weight*Leg length	0.01	−0.01, 0.02	0.01	−0.01, 0.02
HbA_1c_	- Birth weight	−0.02	−0.058, 0.03	−0.03	−0.08, 0.03
	- Leg length	−0.05	−0.11, 0.01	0.04	−0.10, 0.03
	- Birth weight*Leg length	0.01	−0.04, 0.06	0.01	−0.05, 0.06
eGFR	- Birth weight	−5.45	−8.53, −2.38**	−2.95	−5.04, −0.87**
	- Leg length	0.93	−3.97, 2.11	−5.35	−7.89, −2.81**
	- Birth weight*Leg length	−0.52	−4.39, 3.35	−0.30	−2.28, 2.88
GGT	- Birth weight	−3.36	−7.88, 2.33	−3.82	−7.70, 0.06
	- Leg length	−4.92	−10.05, 1.17	−3.32	−8.81, 2.18
	- Birth weight*Leg length	0.12	−3.96, 4.21	−0.48	−4.70, 3.75

* p≤0.05;

** p≤0.001.

Unadjusted: models include sex and birth status (singleton or twin).

Adjusted: models include unadjusted model plus: alcohol, smoking consumption, BMI, physical activity.

The sensitivity analysis using a nine-category version of growth marker mismatches revealed that there were no consistent associations with any of the five individual biomarkers Tables S2a–S2e in File S1).

## Discussion

This study found that mismatches between pre- and post-natal conditions, represented as the interaction between birthweight and leg length in adulthood, were not associated with worse physiological functioning across the circulatory (sBP), respiratory (FEV_1_), metabolic (HbA_1c_), excretory (eGFR) and hepatic (GGT) systems in 36-year-old men and women from the West of Scotland. These findings did not match our hypotheses for sBP, HbA_1c_ or GGT, although they did match our predictions for a lack of association with FEV_1_/eGFR.

The evidence linking mismatches between pre- and post-natal conditions and physiological functioning in early adulthood has received limited attention. Of those studies that have investigated the issue, there have been mixed findings brought about by: using a mixture of definitions or methods for operationalising such mismatches; focusing on disease outcomes; focusing solely on either pre- or post-natal growth; incorrectly analysing the interaction between the pre- and post-natal conditions [27]; testing only one biomarker/physiological system; and/or sampling from older respondents only. A recent meta-analysis found that mismatches between pre- and post-natal growth were associated with increased blood pressure in adulthood [30]. Although there is limited evidence for mismatches between pre- and post-natal conditions, as assessed by growth patterns, being linked with HbA_1c_, they have been linked with increased insulin resistance in 60–79 year-old British women having had low birthweight, but high BMI in childhood [31]. In a study of British middle-aged adults, increased birthweight and trunk length were found to be independently associated with increased FEV_1_, although no interaction was considered [14]. In a study of 60+ year-old British women, increased leg length was associated with lower GGT levels (better functioning). However, to the best of our knowledge, no studies have considered the effects of mismatches between pre- and post-natal growth on GGT. Studies investigating the links between early-life conditions and kidney function/eGFR have focused solely on birthweight due to the major development of the kidney taking place prior to birth, although these have confirmed that lower birthweight is associated with worse kidney outcomes [32]. However, no studies to date have investigated the effects of mismatches between pre- and post-natal growth/conditions on biomarkers across multiple physiological systems.

Our hypothesis that early signs of physiological damage (leading to diagnosable diseases later in life) would already be underway in terms of detrimental effects on sBP, HbA_1c_ and GGT levels in young adults given mismatches between pre- and post-natal conditions in childhood has not been confirmed. This lack of association could be the result of several factors. Firstly, age 36 could be too young to identify significant alterations in these biomarkers. Although most diseases linked to the physiological systems represented in this study (such as diabetes and chronic obstructive pulmonary disease (COPD)) do not manifest themselves until 40+ years of age [33], sBP [34], [35], HbA_1c_ [36], [37] and GGT [38], [39] track well from childhood/adolescence through to adulthood. FEV_1_ [40], [41] and eGFR [42], [43] are also known to track well from early adulthood onwards. Given this lifecourse tracking, we would expect biomarker levels at age 36 to be predictors of biomarker levels (and disease risk) later in adulthood.

Secondly, there is the possibility that there truly is no effect of a mismatch between pre- and post-natal conditions on the biomarkers chosen. It was predicted that for eGFR, and FEV_1_ to a lesser extent, it was unlikely that we would see a significant association with mismatches between pre- and post-natal conditions. However, given the plasticity of GGT in particular, it is surprising that no effect was identified for this biomarker. This study has failed to show any evidence of links between either the pre- or post-natal growth measures with the biomarkers, except for a link between higher birthweight and lower eGFR levels (better kidney function). However, it could be that either pre- or post-natal factors are independent predictors of later outcomes, with the mismatch between the two not being important.

Blood pressure is probably the most-studied of the five biomarkers used here for its association with pre-and post-natal growth conditions. There is good evidence for increased blood pressure in those who have experienced growth mismatches in childhood or adolescence (lower birthweight followed by increased height or weight gain later in life) [30], [44]–[46]. However, there is some evidence that the association between birthweight and blood pressure is not robust to confounding factors and is much weaker than previously thought [47]. Indeed, we found no association between birthweight and blood pressure at age 35. In previous studies, lung function has also been found to be associated with growth/environmental mismatches (catch-up growth from a lower birthweight starting point) [48], [49]. A recent meta-analysis also suggested that a 1 kg increase in birthweight was associated with a moderate increase in FEV_1_ (better lung function) [50]. Similar to blood pressure though, this study found no evidence of such associations. Early life factors relating to diabetes risk later in life have been well studied. [13], [51]–[54]. Forsen et al identified that low birthweight, birth length, ponderal index (mass divided by height^3^) and placenta weight were associated with increased risk of Type II diabetes [13]. Although height and weight at age 7 were not associated with an increased risk of the disease, rapid (catch-up) growth between ages 7 and 15 and more so when birthweight was low. Eriksson et al added to this by showing that rapid increases in BMI in the first 2 years of life were associated with increased risk of diabetes in adulthood (after age 40), although it was larger babies who had experienced slower growth in the first few months of life who had the greatest risk of the disease [52]. Mericq et al investigated the association between small-for-gestational-age (SGA) babies who showed catch-up growth by age 3 and fasting insulin levels at birth and at age 3 [55]. They found that at birth, SGA babies had lower insulin levels compared to average-for-gestational-age (AGA) babies. However, by age 3 (and after catching up to match the AGA babies), SGA babies had significantly higher insulin levels. Again, our study could find no matching associations. Low birthweight has been found to be associated with reduced kidney size in infants and adults [56]–[58]. Schmidt et al showed that weight at 3 and 18 months of age was positively associated with kidney volume, although there was no evidence of mismatches impacting on kidney volume [58]. Animal models of pre/post-natal mismatches in the form of catch-up growth have shown there to be a “second hit” effect of this plasticity on reducing nephron number [59]. Given that kidney development is complete prior to birth, it is not surprising that no association between the mismatch and eGFR was identified here. However, as one would expect, larger birthweight was associated with improved kidney function. Altered liver function has long been proposed as a mechanism by which poor weight gain in utero is associated with increased CHD risk [60]–[62] through increases in serum cholesterol and plasma fibrinogen levels [61], [63]. However, empirical evidence using biomarkers of liver function are lacking.

The third possibility for a lack of association is that more extreme cases of mismatches between pre- and post-natal growth markers are needed to have a measurable effect. Our sensitivity analysis using a categorical variable did not identify any consistent associations with the five biomarkers. This indicates that more extreme mismatches between pre- and post-natal growth are not associated with more detrimental biomarker outcomes. This stayed true even when adjusting for potential confounders, showing the results to be robust.

Finally, a lack of statistical power is a potential issue. However, the sample sizes used here are relatively large and should be suitable for identifying statistically significant findings if they exist. Using MI to address the potential for bias due to missing values did not alter any of the results either. MI (and sample weights) cannot fully address issues of selective drop-out however, so the possibility that those individuals with the greatest growth mismatch and/or biomarker scores are missing from wave 5 of the sample must be considered.

In this study we have used a relatively large, community-based cohort study, with a wide range of biomarkers across key physiological systems. A further strength of the study is that younger respondents at the age of 36 have been included, with most studies typically focusing on middle- and older-aged adults. This has allowed us to focus on physiological measures relatively early on in adulthood and typically before the diagnosis of chronic diseases. It is important that studies aim to identify the pathways and precursors of disease, rather than focus on disease incidence in mid-later life, in order to help identify at-risk individuals prior to the onset of disease. However, our evidence suggests that even at 36 years of age it may be too early to identify physiological consequences of early-life growth patterns (with individual biomarkers at least).

Birthweight is both cheaply and easily measured, as well as being possible to obtain many years after birth. However, it may not represent the best measure for operationalising pre-natal growth, with measures such as body composition and adiposity perhaps more accurate [11], [64], [65]. This study has relied on mothers’ recall of their babies’ birthweight and although not ideal, it has been shown to be comparable to registration data [66]. We also did not have any information regarding gestational age, which could be important in determining if pre-term babies have a distinct link with physiological outcomes in adulthood and if it is these babies who composed our more extreme mismatched group. Leg length represents growth throughout infancy and early childhood [8], [9], so may not be as accurate as a measure of stature/length taken in the first year of life. However, measures of stature were not available for the respondents as infants. Previous studies have typically used BMI or other adiposity measures to assess the interaction between pre- and post-natal conditions. However, leg length can be measured in adulthood as a retrospective measure of post-natal development, especially infant nutrition [10]. In addition, it was not possible to measure BMI in childhood in this sample as the study started when respondents were 15 years old. A trade-off between having biomarker data available in early adulthood and having measures of stature/growth/adiposity during childhood was necessary. The biomarkers chosen were analysed as continuous measures and not converted to clinically relevant (binary) cut-offs. This approach was taken due to the age of the respondents, as most did not show clinically relevant values to justify dichotomising. In addition, it was important for this study that the biomarker measures reflected physiological states prior to the onset of a diagnosable disease in order to help predict disease risk earlier in life.

This study has failed to find a conclusive link between mismatches in pre- and post-natal growth and physiological dysregulation across five key bodily systems. However, this does not mean that growth in childhood is not an important determinant of health later in life. Future work could use models of cumulative physiological burden such as allostatic load that combine information across multiple systems in a synergistic fashion [25]. In addition, studies that have more detailed growth measures taken in childhood could also provide more robust indicators of growth and plasticity than achieved here.

## Supporting Information

Checklist S1 STROBE Statement: Checklist of items that should be included in reports of cohort. studies
(DOCX)

File S1 Contains Tables S1–S2e. Table S1, Regression results for the five biomarkers regressed against birthweight and leg length (complete-case analysis). Table S2a, Regression results for systolic blood pressure (sBP) by a categorical growth pattern construct. Table S2b, Regression results for Forced Expiratory Volume in one second (FEV_1_) by a categorical growth pattern construct. Table S2c, Regression results for glycated haemoglobin (HbA_1c_) by a categorical growth pattern construct. Table S2d, Regression results for estimated Glomerular Filtration Rate (eGFR) by a categorical growth pattern construct. Table S2e, Regression results for gamma-glutamyltransferase (GGT) by a categorical growth pattern construct.
(DOC)

## References

[1] Byrne CD, Phillips DI (2000) Fetal origins of adult disease: epidemiology and mechanisms. J Clin Pathol 53: 822–828. 11127263 10.1136/jcp.53.11.822 PMC1731115

[2] Kramer MS (1987) Determinants of low birth weight: methodological assessment and meta-analysis. Bull World Health Organ 65: 663–737. 3322602 PMC2491072

[3] Lewit EM, Kerrebrock N (1997) Population-based growth stunting. Future Child 7: 149–156. 9299843

[4] Pike KC, Hanson MA, Godfrey KM (2008) Developmental mismatch: consequences for later cardiorespiratory health. BJOG 115: 149–157. 18081597 10.1111/j.1471-0528.2007.01603.x

[5] Barker DJ (2004) Developmental origins of adult health and disease. J Epidemiol Community Health 58: 114–115. 14729887 10.1136/jech.58.2.114 PMC1732687

[6] Bateson P, Barker D, Clutton-Brock T, Deb D, D’Udine B, et al (2004) Developmental plasticity and human health. Nature 430: 419–421. 15269759 10.1038/nature02725

[7] Bateson P, Gluckman P (2012) Plasticity and robustness in development and evolution. Int J Epidemiol 41: 219–223. 22422456 10.1093/ije/dyr240

[8] Gunnell DJ, Smith GD, Frankel SJ, Kemp M, Peters TJ (1998) Socio-economic and dietary influences on leg length and trunk length in childhood: a reanalysis of the Carnegie (Boyd Orr) survey of diet and health in prewar Britain (1937–39). Paediatr Perinat Epidemiol 12 Suppl 196–113. 9690276 10.1046/j.1365-3016.1998.0120s1096.x

[9] Wadsworth ME, Hardy RJ, Paul AA, Marshall SF, Cole TJ (2002) Leg and trunk length at 43 years in relation to childhood health, diet and family circumstances; evidence from the 1946 national birth cohort. Int J Epidemiol 31: 383–390. 11980800

[10] Lawlor DA, Davey Smith G, Ebrahim S (2003) Association between leg length and offspring birthweight: partial explanation for the trans-generational association between birthweight and cardiovascular disease: findings from the British Women’s Heart and Health Study. Paediatr Perinat Epidemiol 17: 148–155. 12675781 10.1046/j.1365-3016.2003.00479.x

[11] Barker DJ, Eriksson JG, Forsen T, Osmond C (2002) Fetal origins of adult disease: strength of effects and biological basis. Int J Epidemiol 31: 1235–1239. 12540728 10.1093/ije/31.6.1235

[12] Singhal A, Lucas A (2004) Early origins of cardiovascular disease: is there a unifying hypothesis? Lancet 363: 1642–1645. 15145640 10.1016/S0140-6736(04)16210-7

[13] Forsen T, Eriksson J, Tuomilehto J, Reunanen A, Osmond C, et al (2000) The fetal and childhood growth of persons who develop type 2 diabetes. Ann Intern Med 133: 176–182. 10906831 10.7326/0003-4819-133-3-200008010-00008

[14] Orfei L, Strachan DP, Rudnicka AR, Wadsworth ME (2008) Early influences on adult lung function in two national British cohorts. Arch Dis Child 93: 570–574. 17626144 10.1136/adc.2006.112201

[15] Stein CE, Kumaran K, Fall CH, Shaheen SO, Osmond C, et al (1997) Relation of fetal growth to adult lung function in south India. Thorax 52: 895–899. 9404378 10.1136/thx.52.10.895 PMC1758421

[16] Barker DJ (1993) Fetal origins of coronary heart disease. Br Heart J 69: 195–196. 8461215 10.1136/hrt.69.3.195 PMC1024978

[17] Luyckx VA, Bertram JF, Brenner BM, Fall C, Hoy WE, et al (2013) Effect of fetal and child health on kidney development and long-term risk of hypertension and kidney disease. The Lancet 382: 273–283. 10.1016/S0140-6736(13)60311-6 23727166

[18] Benzeval M, Der G, Ellaway A, Hunt K, Sweeting H, et al (2009) Cohort Profile: West of Scotland Twenty-07 Study: Health in the Community. Int J Epidemiol 38: 1215–1223. 18930962 10.1093/ije/dyn213 PMC2935558

[19] Cole TJ (1975) Linear and Proportional Regression Models in the Prediction of Ventilatory Function. Journal of the Royal Statistical Society Series A (General) 138: 297–338.

[20] Cockcroft DW, Gault MH (1976) Prediction of creatinine clearance from serum creatinine. Nephron 16: 31–41. 1244564 10.1159/000180580

[21] Law MR, Wald NJ, Morris JK, Jordan RE (2003) Value of low dose combination treatment with blood pressure lowering drugs: analysis of 354 randomised trials. BMJ 326: 1427. 12829555 10.1136/bmj.326.7404.1427 PMC162261

[22] Kinshuck D, Lamb P, Griffiths U (2011) Control of Type 2 diabetes. Birmingham, UK.

[23] Howe LD, Tilling K, Galobardes B, Smith GD, Gunnell D, et al (2012) Socioeconomic differences in childhood growth trajectories: at what age do height inequalities emerge? Journal of Epidemiology and Community Health 66: 143–148. 20724285 10.1136/jech.2010.113068 PMC3245896

[24] Silva LM, van Rossem L, Jansen PW, Hokken-Koelega ACS, Moll HA, et al (2012) Children of Low Socioeconomic Status Show Accelerated Linear Growth in Early Childhood; Results from the Generation R Study. PLoS One 7: e37356. 22649522 10.1371/journal.pone.0037356 PMC3359354

[25] Seeman T, Epel E, Gruenewald T, Karlamangla A, McEwen BS (2010) Socio-economic differentials in peripheral biology: cumulative allostatic load. Ann N Y Acad Sci 1186: 223–239. 20201875 10.1111/j.1749-6632.2009.05341.x

[26] Seaman S, Benzeval M (2011) The West of Scotland Twenty-07 Study: Inverse probability weights for Wave 5: MRC/CSO Social and Public Health Sciences Unit Working Paper 27, Glasgow.

[27] Lucas A, Fewtrell MS, Cole TJ (1999) Fetal origins of adult disease-the hypothesis revisited. BMJ 319: 245–249. 10417093 10.1136/bmj.319.7204.245 PMC1116334

[28] Bonellie S (2005) New centile charts for birthweights in Scotland. Edinburgh: NHS Scotland.

[29] ScottishGovernment (2012) The Scottish Health Survey 2011: Volume 1 - Adults. Edinburgh: The Scottish Government.

[30] Huxley RR, Shiell AW, Law CM (2000) The role of size at birth and postnatal catch-up growth in determining systolic blood pressure: a systematic review of the literature. Journal of Hypertension 18: 815–831. 10930178 10.1097/00004872-200018070-00002

[31] Lawlor DA, Davey Smith G, Ebrahim S (2003) Life Course Influences on Insulin Resistance: Findings from the British Women’s Heart and Health Study. Diabetes Care 26: 97–103. 12502664 10.2337/diacare.26.1.97

[32] White SL, Perkovic V, Cass A, Chang CL, Poulter NR, et al (2009) Is low birth weight an antecedent of CKD in later life? A systematic review of observational studies. American journal of kidney diseases : the official journal of the National Kidney Foundation 54: 248–261. 19339091 10.1053/j.ajkd.2008.12.042

[33] NHLBI (2012) USA’s National Heart, Lung and Blood Institute - Who Is at Risk for COPD?

[34] Wills AK, Lawlor DA, Matthews FE, Sayer AA, Bakra E, et al (2011) Life course trajectories of systolic blood pressure using longitudinal data from eight UK cohorts. PLoS Med 8: e1000440. 21695075 10.1371/journal.pmed.1000440 PMC3114857

[35] Lawlor DA, Davey Smith G (2007) Early-Llife Influences on Blood Pressure. In: Lip YH, Hall JE, editors. Comprehensive Hypertension. Philadelphia, PA: Mosby Elsevier. 41–49.

[36] Higgins T, Cembrowski G, Tran D, Lim E, Chan J (2009) Influence of variables on hemoglobin A1c values and nonheterogeneity of hemoglobin A1c reference ranges. J Diabetes Sci Technol 3: 644–648. 20144306 10.1177/193229680900300404 PMC2769978

[37] Pani LN, Korenda L, Meigs JB, Driver C, Chamany S, et al (2008) Effect of aging on A1C levels in individuals without diabetes: evidence from the Framingham Offspring Study and the National Health and Nutrition Examination Survey 2001–2004. Diabetes Care 31: 1991–1996. 18628569 10.2337/dc08-0577 PMC2551641

[38] Puukka K, Hietala J, Koivisto H, Anttila P, Bloigu R, et al (2006) Age-related changes on serum ggt activity and the assessment of ethanol intake. Alcohol Alcohol 41: 522–527. 16855003 10.1093/alcalc/agl052

[39] Schiele F, Guilmin AM, Detienne H, Siest G (1977) Gamma-glutamyltransferase activity in plasma: statistical distributions, individual variations, and reference intervals. Clin Chem 23: 1023–1028. 15742

[40] Janssens J, Pache J, Nicod L (1999) Physiological changes in respiratory function associated with ageing. European Respiratory Journal 13: 197–205. 10836348 10.1034/j.1399-3003.1999.13a36.x

[41] Kerstjens HA, Rijcken B, Schouten JP, Postma DS (1997) Decline of FEV1 by age and smoking status: facts, figures, and fallacies. Thorax 52: 820–827. 9371217 10.1136/thx.52.9.820 PMC1758654

[42] Glassock RJ, Winearls C (2009) Ageing and the glomerular filtration rate: truths and consequences. Trans Am Clin Climatol Assoc 120: 419–428. 19768194 PMC2744545

[43] Lindeman RD, Tobin J, Shock NW (1985) Longitudinal studies on the rate of decline in renal function with age. J Am Geriatr Soc 33: 278–285. 3989190 10.1111/j.1532-5415.1985.tb07117.x

[44] Lawlor DA, Smith GD (2005) Early life determinants of adult blood pressure. Curr Opin Nephrol Hypertens 14: 259–264. 15821420 10.1097/01.mnh.0000165893.13620.2b

[45] Langenberg C, Shipley MJ, Batty GD, Marmot MG (2005) Adult socioeconomic position and the association between height and coronary heart disease mortality: findings from 33 years of follow-up in the Whitehall Study. Am J Public Health 95: 628–632. 15798120 10.2105/2004.046219 PMC1449231

[46] Horta BL, Barros FC, Victora CG, Cole TJ (2003) Early and late growth and blood pressure in adolescence. Journal of Epidemiolgy and Community Health 57: 226–230. 10.1136/jech.57.3.226 PMC1732403 12594200

[47] Huxley R, Neil A, Collins R (2002) Unravelling the fetal origins hypothesis: is there really an inverse association between birthweight and subsequent blood pressure? Lancet 360: 659–665. 12241871 10.1016/S0140-6736(02)09834-3

[48] Hancox RJ, Poulton R, Greene JM, McLachlan CR, Pearce MS, et al (2009) Associations between birth weight, early childhood weight gain and adult lung function. Thorax 64: 228–232. 19052051 10.1136/thx.2008.103978

[49] Canoy D, Pekkanen J, Elliott P, Pouta A, Laitinen J, et al (2007) Early growth and adult respiratory function in men and women followed from the fetal period to adulthood. Thorax 62: 396–402. 17105780 10.1136/thx.2006.066241 PMC2117170

[50] Lawlor DA, Ebrahim S, Davey Smith G (2005) Association of birth weight with adult lung function: findings from the British Women’s Heart and Health Study and a meta-analysis. Thorax 60: 851–858. Epub 2005 Jul 2029. 10.1136/thx.2005.042408 PMC1747204 16055617

[51] Barker DJ, Hales CN, Fall CH, Osmond C, Phipps K, et al (1993) Type 2 (non-insulin-dependent) diabetes mellitus, hypertension and hyperlipidaemia (syndrome X): relation to reduced fetal growth. Diabetologia 36: 62–67. 8436255 10.1007/BF00399095

[52] Eriksson JG, Forsen TJ, Osmond C, Barker DJ (2003) Pathways of infant and childhood growth that lead to type 2 diabetes. Diabetes Care 26: 3006–3010. 14578231 10.2337/diacare.26.11.3006

[53] Hales CN, Barker DJ (1992) Type 2 (non-insulin-dependent) diabetes mellitus: the thrifty phenotype hypothesis. Diabetologia 35: 595–601. 1644236 10.1007/BF00400248

[54] Hales CN, Barker DJ, Clark PM, Cox LJ, Fall C, et al (1991) Fetal and infant growth and impaired glucose tolerance at age 64. BMJ 303: 1019–1022. 1954451 10.1136/bmj.303.6809.1019 PMC1671766

[55] Mericq V, Ong KK, Bazaes R, Pena V, Avila A, et al (2005) Longitudinal changes in insulin sensitivity and secretion from birth to age three years in small- and appropriate-for-gestational-age children. Diabetologia 48: 2609–2614. 16283238 10.1007/s00125-005-0036-z

[56] Singh GR, Hoy WE (2004) Kidney volume, blood pressure, and albuminuria: findings in an Australian aboriginal community. Am J Kidney Dis 43: 254–259. 14750090 10.1053/j.ajkd.2003.10.015

[57] Spencer J, Wang Z, Hoy W (2001) Low birth weight and reduced renal volume in Aboriginal children. Am J Kidney Dis 37: 915–920. 11325672 10.1016/s0272-6386(05)80006-x

[58] Schmidt IM, Chellakooty M, Boisen KA, Damgaard IN, Mau Kai C, et al (2005) Impaired kidney growth in low-birth-weight children: distinct effects of maturity and weight for gestational age. Kidney Int 68: 731–740. 16014050 10.1111/j.1523-1755.2005.00451.x

[59] Boubred F, Daniel L, Buffat C, Feuerstein JM, Tsimaratos M, et al.. (2009) Early postnatal overfeeding induces early chronic renal dysfunction in adult male rats. Am J Physiol Renal Physiol 297: F943–951. Epub 2009 Aug 2005. 10.1152/ajprenal.90704.2008 19656908

[60] Barker DJ (2002) Fetal programming of coronary heart disease. Trends Endocrinol Metab 13: 364–368. 12367816 10.1016/s1043-2760(02)00689-6

[61] Barker DJ, Martyn CN, Osmond C, Hales CN, Fall CH (1993) Growth in utero and serum cholesterol concentrations in adult life. BMJ 307: 1524–1527. 8274921 10.1136/bmj.307.6918.1524 PMC1679540

[62] Fall C (1992) Nutrition in early life and later outcome. Eur J Clin Nutr 46 Suppl 4S57–63. 1286650

[63] Barker DJ, Meade TW, Fall CH, Lee A, Osmond C, et al (1992) Relation of fetal and infant growth to plasma fibrinogen and factor VII concentrations in adult life. BMJ 304: 148–152. 1737158 10.1136/bmj.304.6820.148 PMC1881173

[64] Leon DA (2004) Biological theories, evidence, and epidemiology. Int J Epidemiol 33: 1167–1171. 15567870 10.1093/ije/dyh389

[65] Robinson R (2001) The fetal origins of adult disease. BMJ 322. 10.1136/bmj.322.7283.375 PMC1119617 11179140

[66] Tate AR, Dezateux C, Cole TJ, Davidson L (2005) Factors affecting a mother’s recall of her baby’s birth weight. Int J Epidemiol 34: 688–695. 15737964 10.1093/ije/dyi029

