# Supplementary material for: Do Mismatches between Pre- and Post-Natal Environments Influence Adult Physiological Functioning?
Source: PLoS One. 2014 Jan 31;9(1):e86953. doi: 10.1371/journal.pone.0086953 (PMC3908925; doi:10.1371/journal.pone.0086953)
Supplement: File S1 — Contains Tables S1–S2e. Table S1, Regression results for the five biomarkers regressed against birthweight and leg length (complete-case analysis). Table S2a, Regression results for systolic blood pressure (sBP) by a categorical growth pattern construct. Table S2b, Regression results for Forced Expiratory Volume in one second (FEV1) by a categorical growth pattern construct. Table S2c, Regression results for glycated haemoglobin (HbA1c) by a categorical growth pattern construct. Table S2d, Regression results for estimated Glomerular Filtration Rate (eGFR) by a categorical growth pattern construct. Table S2e, Regression results for gamma-glutamyltransferase (GGT) by a categorical growth pattern construct. (DOC) [file pone.0086953.s002.doc]

**Table S1** Regression results for the five biomarkers regressed against birthweight and leg length (complete-case analysis)

|  |  |  | | | |
| --- | --- | --- | --- | --- | --- |
|  |  | **Unadjusted** | | **Adjusted** | |
|  |  | **β** | **95% CI** | **β** | **95% CI** |
|  |  |  |  |  |  |
| BP (n=768) | - Birth weight | -0.34 | -1.49, 0.80 | -0.87 | -1.97, 0.23 |
|  | - Leg length | -0.45 | -2.18, 1.28 | 0.55 | -1.01, 2.12 |
|  | - Birth weight*Leg length | 1.01 | 0.09, 1.93* | 0.46 | -0.34, 1.26 |
|  |  |  |  |  |  |
| FEV_1_  (n=749) | - Birth weight | -0.01 | -0.03, 0.01 | -0.01 | -0.03, 0.01 |
|  | - Leg length | 0.01 | -0.01, 0.03 | 0.01 | -0.01, 0.03 |
|  | - Birth weight*Leg length | 0.01 | -0.01, 0.02 | 0.01 | -0.01, 0.02 |
|  |  |  |  |  |  |
| HbA_1c_  (n=644) | - Birth weight | -0.01 | -0.06, 0.04 | -0.01 | -0.03, 0.01 |
|  | - Leg length | -0.05 | -0.11, 0.01 | 0.01 | -0.01, 0.03 |
|  | - Birth weight*Leg length | 0.01 | -0.03, 0.03 | 0.01 | -0.01, 0.02 |
|  |  |  |  |  |  |
| eGFR  (n=643) | - Birth weight | -5.48 | -8.75, -2.22** | -3.55 | -5.63, -1.47** |
|  | - Leg length | 0.66 | -2.38, 3.71 | -4.62 | -6. 98, -2.35** |
|  | - Birth weight*Leg length | -1.85 | -6.17, 2.46 | 0.47 | -2.01, 2.94 |
|  |  |  |  |  |  |
| GGT  (n=643) | - Birth weight | -2.44 | -6.68, 1.81 | -3.63 | -7.29, 0.03 |
|  | - Leg length | -3.85 | -8.22, 0.53 | -1.68 | -6.38, 3.02 |
|  | - Birth weight*Leg length | -0.05 | -2.43, 2.33 | -1.43 | -4.72, 1.85 |
|  |  |  |  |  |  |

*p≤0.05; **p≤0.001

Unadjusted: models include sex and birth status (singleton or twin)

Adjusted: models include unadjusted models plus: alcohol, smoking consumption, BMI, physical activity, childhood SEP

**Table S2a** Regression results for systolic blood pressure (sBP) by a categorical growth pattern construct

| **sBP** | **Sample Size** | **Unadjusted** | | **Adjusted** | |
| --- | --- | --- | --- | --- | --- |
|  |  | **β** | **95% CI** | **β** | **95% CI** |
|  |  |  |  |  |  |
| Low birthweight – Small leg length | 36 | -1.10 | -8.57, 6.36 | -1.43 | -9.22, 6.36 |
| Low birthweight – Medium leg length | 22 | 1.52 | -7.96, 11.00 | 0.72 | -9.16, 10.60 |
| **Low birthweight – Long leg length** | **12** | **0 (ref)** |  | **0 (ref)** | **-** |
| Average birthweight – Small leg length | 232 | -1.69 | -8.55, 5.18 | -2.70 | -9.84, 4.44 |
| Average birthweight – Medium leg length | 225 | 0.68 | -5.93, 7.29 | 0.79 | -6.19, 7.77 |
| Average birthweight – Long leg length | 226 | -2.57 | -9.52, 4.37 | -1.64 | -9.11, 5.84 |
| High birthweight – Small leg length | 21 | 0.60 | -8.23, 9.44 | 1.70 | -10.22, 6.83 |
| High birthweight – Medium leg length | 40 | -4.76 | -12.77, 3.25 | -5.50 | -14.15, 3.14 |
| High birthweight – Long leg length | 47 | -0.63 | -8.78, 7.52 | -1.56 | -9.82, 6.71 |
|  |  |  |  |  |  |

* p≤0.05

**Table S2b** Regression results for Forced Expiratory Volume in one second (FEV_1_) by a categorical growth pattern construct

| **FEV_1_** | **Sample Size** | **Unadjusted** | | **Adjusted** | |
| --- | --- | --- | --- | --- | --- |
|  |  | **β** | **95% CI** | **β** | **95% CI** |
|  |  |  |  |  |  |
| Low birthweight – Small leg length | 33 | -0.01 | -1.13, 0.11 | -0.01 | -0.13, 0.11 |
| Low birthweight – Medium leg length | 22 | 0.01 | -0.11, 0.13 | 0.01 | -0.12, 0.13 |
| **Low birthweight – Long leg length** | **11** | **0 (ref)** | **-** | **0 (ref)** | **-** |
| Average birthweight – Small leg length | 222 | -0.04 | -0.14, 0.06 | -0.05 | -0.15, 0.05 |
| Average birthweight – Medium leg length | 215 | -0.04 | -0.14, 0.06 | -0.04 | -0.13, 0.07 |
| Average birthweight – Long leg length | 217 | -0.03 | -0.13, 0.07 | -0.03 |  |
| High birthweight – Small leg length | 21 | 0.01 | -0.11, 0.12 | -0.01 | -0.13, 0.10 |
| High birthweight – Medium leg length | 39 | -0.15 | -0.28, -0.01* | -0.15 | -0.29, -0.01* |
| High birthweight – Long leg length | 44 | -0.06 | -0.19, 0.08 | -0.05 | -0.18, 0.08 |
|  |  |  |  |  |  |

* p≤0.05

**Table S2c** Regression results for glycated haemoglobin (HbA_1c_) by a categorical growth pattern construct

| **HbA_1c_** | **Samples Size** | **Unadjusted** | | **Adjusted** | |
| --- | --- | --- | --- | --- | --- |
|  |  | **β** | **95% CI** | **β** | **95% CI** |
|  |  |  |  |  |  |
| Low birthweight – Small leg length | 29 | 0.52 | 0.01, 1.03 | 0.51 | 0.02, 1.00 |
| Low birthweight – Medium leg length | 20 | 0.19 | -0.17, 0.54 | 0.17 | -0.17, 0.52 |
| **Low birthweight – Long leg length** | **8** | **0 (ref)** | **-** | **0 (ref)** | **-** |
| Average birthweight – Small leg length | 190 | 0.16 | -0.11, 0.42 | 0.14 | -0.12, 0.40 |
| Average birthweight – Medium leg length | 186 | 0.15 | -0.11, 0.41 | 0.15 | -0.10, 0.41 |
| Average birthweight – Long leg length | 186 | 0.13 | -0.12, 0.38 | 0.14 | -0.12, 0.39 |
| High birthweight – Small leg length | 19 | 0.63 | 0.01, 1.25 | 0.60 | -0.01, 1.22 |
| High birthweight – Medium leg length | 31 | 0.05 | -0.23, 0.33 | 0.04 | -0.24, 0.32 |
| High birthweight – Long leg length | 40 | 0.09 | -0.17, 0.35 | 0.08 | -0.17, 0.33 |
|  |  |  |  |  |  |

* p≤0.05

**Table S2d** Regression results for estimated Glomerular Filtration Rate (eGFR) by a categorical growth pattern construct

| **eGFR** | **Sample Size** | **Unadjusted** | | **Adjusted** | |
| --- | --- | --- | --- | --- | --- |
|  |  | **β** | **95% CI** | **β** | **95% CI** |
|  |  |  |  |  |  |
| Low birthweight – Small leg length | 29 | 5.81 | -20.29, 31.92 | 8.55 | -9.52, 26.62 |
| Low birthweight – Medium leg length | 20 | 5.56 | -20.42, 31.53 | 9.47 | -7.13, 26.07 |
| **Low birthweight – Long leg length** | **9** | **0 (ref)** | **-** | **0 (ref)** | **-** |
| Average birthweight – Small leg length | 189 | 4.62 | -16.78, 26.01 | 7.99 | -5.93, 21.92 |
| Average birthweight – Medium leg length | 185 | 3.49 | -19.21, 26.20 | 3.93 | -11.27, 19.14 |
| Average birthweight – Long leg length | 186 | 1.39 | -20.07, 22.84 | -2.16 | -16.32, 12.00 |
| High birthweight – Small leg length | 19 | -29.42 | -58.54, -0.31* | -15.30 | -35.07, 4.46 |
| High birthweight – Medium leg length | 31 | -2.77 | -28.12, 22.58 | 3.82- | -13.63, 21.26 |
| High birthweight – Long leg length | 40 | -14.87 | -39.50, 9.76 | 12.44 | -29.03, 4.14 |
|  |  |  |  |  |  |

* p≤0.05

**Table S2e** Regression results for gamma-glutamyltransferase (GGT) by a categorical growth pattern construct

| **GGT** | **Sample Size** | **Unadjusted** | | **Adjusted** | |
| --- | --- | --- | --- | --- | --- |
|  |  | **β** | **95% CI** | **β** | **95% CI** |
|  |  |  |  |  |  |
| Low birthweight – Small leg length | 29 | 24.30 | -11.75, 60.35 | 22.92 | -11.19, 57.03 |
| Low birthweight – Medium leg length | 20 | 13.10 | -17.37, 43.57 | 13.66 | -17.03, 44.36 |
| **Low birthweight – Long leg length** | **9** | **0 (ref)** | **-** | **0 (ref)** | **-** |
| Average birthweight – Small leg length | 189 | 13.57 | -8.00, 35.13 | 10.36 | -11.82, 32.54 |
| Average birthweight – Medium leg length | 185 | 11.66 | -7.64, 30.96 | 10.80 | -9.21, 30.82 |
| Average birthweight – Long leg length | 186 | 5.24 | -13.74, 24.22 | 5.17 | -15.32, 25.65 |
| High birthweight – Small leg length | 19 | 25.63 | -9.23, 60.48 | 19.91 | -13.92, 53.74 |
| High birthweight – Medium leg length | 32 | 13.77 | -10.62, 38.16 | 10.40 | -14.70, 35.49 |
| High birthweight – Long leg length | 40 | -5.40 | -24.53, 13.73 | -7.25 | -29.15, 14.64 |
|  |  |  |  |  |  |
